# Anti-high mobility group box-1 monoclonal antibody treatment provides protection against influenza A virus (H1N1)-induced pneumonia in mice

**DOI:** 10.1186/s13054-015-0983-9

**Published:** 2015-06-11

**Authors:** Nobuyuki Nosaka, Masato Yashiro, Mutsuko Yamada, Yosuke Fujii, Hirokazu Tsukahara, Keyue Liu, Masahiro Nishibori, Akihiro Matsukawa, Tsuneo Morishima

**Affiliations:** Department of Pediatrics, Okayama University Graduate School of Medicine, Dentistry and Pharmaceutical Sciences, Okayama, Japan; Department of Pharmacology, Okayama University Graduate School of Medicine, Dentistry and Pharmaceutical Sciences, Okayama, Japan; Department of Pathology and Experimental Medicine, Okayama University Graduate School of Medicine, Dentistry and Pharmaceutical Sciences, Okayama, Japan

## Abstract

**Introduction:**

Provision for the emergence of an influenza pandemic is an urgent issue. The discovery of a novel anti-influenza therapeutic approach would increase the effectiveness of traditional virus-based strategies. This study was undertaken to evaluate the therapeutic effects of anti-high mobility group box-1 (HMGB1) monoclonal antibody (mAb) treatment on influenza A virus (H1N1)-induced pneumonia in mice.

**Methods:**

Nine-week-old male C57BL/6 mice were inoculated with H1N1, then anti-HMGB1 mAb or control mAb were administered intravenously at 1, 24 and 48 hours after H1N1 inoculation and the survival rate was analyzed. Lung lavage and histopathological analysis were performed on days 3, 5, 7 and 10 after inoculation.

**Results:**

Anti-HMGB1 mAb significantly improved the survival rate of H1N1-inoculated mice (1 out of 15 versus 8 out of 15 deaths in the anti-HMGB1 mAb-treated group versus the control mAb-treated group, *p* < 0.01), although the treatment did not affect virus propagation in the lungs. The treatment also significantly attenuated histological changes and neutrophil infiltration in the lungs of H1N1-inoculated mice. This was associated with inhibition of HMGB1 and suppression of inflammatory cytokine/chemokine expression and oxidative stress enhancement, which were observed in H1N1-inoculated mice. The expression of receptor for advanced glycation end products and nuclear factor κB was attenuated by the treatment.

**Conclusions:**

Anti-HMGB1 mAb may provide a novel and effective pharmacological strategy for severe influenza virus infection in humans by reducing the inflammatory responses induced by HMGB1.

**Electronic supplementary material:**

The online version of this article (doi:10.1186/s13054-015-0983-9) contains supplementary material, which is available to authorized users.

## Introduction

The first influenza pandemic of this century, the 2009 H1N1 pandemic, has taught us many lessons [1]. The 2009 A (H1N1) influenza virus was a relatively mild pathogen for the majority of patients, although up to 20 % of patients developed progressive, severe H1N1-induced pneumonia requiring hospitalization [2–4]. The next influenza pandemic is predicted to arise in the near future. Tremendous advances have been made in the development of anti-influenza drugs in the last few decades [5, 6]; however, their therapeutic effects are not guaranteed because of the need to administer these agents early after onset, and the emergence of resistant virus strains [7, 8]. Additionally, the protective effects and the production and availability of influenza vaccines are also limited [9]. This situation underlies the pressing need to define novel therapeutic targets involved in disease pathogenesis and progression.

Excessive cytokine production is considered to be a key contributor to the pathophysiology of severe influenza infection [10]. Shi et al. reported inhibition of tumor necrosis factor-alpha (TNF-α) with etanercept, an agent that provided protection against H1N1 infection in mice [11]. The inhibition of an inflammatory cytokine, therefore, represented a promising novel strategy against influenza infection. In this study, we focused on high mobility group box-1 (HMGB1), originally identified as a ubiquitous DNA-binding protein [12], which is now also recognized as a damage-associated molecular pattern molecule [13]. HMGB1 has been proposed to be a crucial mediator in the pathogenesis of many diseases, including sepsis [14], autoimmunity [15], acute lung inflammation [16] and several severe viral infections [17–19]. HMGB1 can be released passively from necrotic cells and/or actively secreted by macrophages or monocytes into the extracellular milieu [20]. Extracellular HMGB1 can elicit the production of proinflammatory cytokines that induce inflammatory responses through several immune receptors, including the toll-like receptor 4 (TLR4) [21] and the receptor for advanced glycation end product (RAGE) [22, 23]. Moreover, intranuclear HMGB1 has also been reported to play a significant role in the replication of influenza viruses [24].

Recently, we found that anti-HMGB1 monoclonal antibody (mAb) markedly inhibited fluid percussion-induced brain edema in rats by inhibiting HMGB1 translocation [25]. These results prompted us to evaluate the therapeutic effects of anti-HMGB1 mAb administration in severe pneumonia induced by influenza virus in anticipation of development of the drug. Here we provide compelling data demonstrating that anti-HMGB1 mAb may provide a novel and effective pharmacological therapeutic strategy for severe influenza virus infection by reducing the inflammatory responses induced by HMGB1.

## Methods

### Ethics

This study was approved by the Animal Use Committee of Okayama University Graduate School of Medicine, Dentistry and Pharmaceutical Sciences (No. OKU-2014502) and was conducted in accordance with National Institutes of Health Guidelines.

### Experimental animals and establishment of an influenza virus-induced pneumonia model

Eight-week-old male C57BL/6 mice (21–24 g body weight) were purchased from Charles River Laboratories (Yokohama, Japan). They were housed in a specific-pathogen-free animal facility at 25 °C with a 12-hr light/dark cycle and fed a standard pellet diet (Oriental MF; Oriental Yeast Ltd., Tokyo, Japan).

Influenza virus A/Puerto Rico/8/34 (H1N1), a mouse-adapted strain, was used throughout the study. The virus was propagated in 10-day-old embryonated chicken eggs. The virus titer was quantitated by a plaque assay using Madin–Darby canine kidney cells and its 50 % mouse lethal dose (MLD_50_) was 100 plaque-forming units (pfu). Nine-week-old mice were anesthetized by intraperitoneal injection of ketamine (50 mg/kg) and pentobarbital (30 mg/kg). They were then inoculated intranasally with 100 pfu (1 MLD_50_) of H1N1 suspended in 25 μL of sterile phosphate-buffered saline. The animals were allowed to recover and analyzed as described below. The day of virus inoculation was defined as day 0.

### Administration of anti-HMGB1 mAb to H1N1-inoculated mice

The mice were randomly assigned to two groups after virus inoculation, and an anti-HMGB1 mAb (#10-22, immunoglobulin G_2a_ subclass, 2 mg/kg) [26] or class-matched control mAb (anti-*Keyhole Limpet* hemocyanin) was administered intravenously via the caudal vein at 1, 24 and 48 hr after virus inoculation. These mAb were produced by our group as described previously [26].

The dose of anti-HMGB1 mAb (2 mg/kg/mouse) was considered sufficient, because a larger dose (4 mg/kg/mouse) did not further reduce the levels of HMGB1 and cytokines in the lungs. We injected anti-HMGB1 mAb in triplicate after virus inoculation, as the levels of HMGB1 remained elevated during the observation period (10 days).

### Survival rate analysis

Survival was observed until day 28 (15 mice per group). No other parameters were measured in the mice.

### Pathological analysis

Pathological analyses were performed on days 3, 5, 7 and 10 after H1N1 inoculation (10 mice per group at each time point). The mice were humanely euthanized and their blood and bronchoalveolar lavage fluid (BALF) was sampled for measurement of cytokines, chemokines and hydroperoxides. The surgical procedures for pathological analysis and lung histological examination were performed as described previously [27]. Immunohistochemical analysis was performed using an antibody against granulocyte-differentiation antigen (BioLegend, San Diego, CA, USA) [28] to detect neutrophil infiltration into the lung according to the manufacturer’s instructions.

The lung injury score was calculated as previously described [29]. Briefly, four readily identifiable pathological processes were graded semiquantitatively on a scale of 0 to 4: alveolar and interstitial edema, hemorrhage, margination and infiltration of inflammatory cells, and formation of bronchiolitis. A score of 0 represented normal lung, 1 represented mild, 2 was moderate, 3 was severe, and 4 denoted very severe changes. For each mouse, the lung injury score was calculated by adding the individual grades (the mean value of five sections) for each category. The histology was reviewed by two pathologists in a blinded manner (NN and SF).

Bronchoalveolar lavage was performed as previously described [27]. Briefly, the right lung was lavaged with 1 mL of cold phosphate-buffered saline. The recovered BALF was collected and centrifuged, and the supernatant was stored at −80 °C prior to cytokine analysis. The total cell number in the BALF was calculated from the cell number in 200 μL of sediment. The percentage of neutrophils was determined and the total neutrophil number in the BALF was calculated and expressed per animal.

### Real-time polymerase chain reaction (PCR)

Total RNA was extracted from the middle portion of the left lung using RNeasy Plus Mini (Qiagen, Hilden, Germany). Total RNA was reverse-transcribed to cDNA using RETROscript (Applied Biosystems, Foster City, CA, USA) according to the manufacturer’s instructions. Briefly, 1 μg total RNA was combined with random decamers and heated to 75 °C for 3 minutes. The RNA-random decamer mixture was combined with reverse transcriptase buffer, dNTP mix, RNase inhibitor and Moloney murine leukemia virus reverse transcriptase. The RNA was reverse-transcribed at 43 °C for 60 minutes, and the enzyme was inactivated at 92 °C for 10 minutes. The cDNA was used as a template for PCR using the 7500 Real-Time PCR System (Applied Biosystems).

The probe and primers for the analysis of the expression of influenza virus type A (M gene) mRNA were as follows: TaqMan probe, 5′-6CCCTCAAAGCCGAGATCGCACAGAGAC-3′; forward primer, 5′-CGTTCTCTCTATCATCCCGTCAG-3′; reverse primer, 5′-GGTCTTGTCTTTAGCCATTCCATG-3′ [GenBank NC_002016]. For analysis of signaling pathways, we performed real-time PCR with the SYBR Premix Ex *Taq* (Takara Biomedicals, Shiga, Japan) according to the manufacturer’s protocol. The sense and antisense primers used for analysis of the expression of mRNA were as follows: glyceralaldehyde-3-phosphate dehydrogenase (GAPDH), 5′-TGACGTGCCGCCTGGAGAAA-3′ and 5′-AGTGTAGCCCAAGATGCCCTTCAG-3′ [GenBank NM_008084]; RAGE, 5′-CTAGAGCCTGGGTGCTGGTTC-3′ and 5′-GTTTCCATTCTAGCTGCTGGGGC-3′ [GenBank NM_007425]; NF-κB (p65), 5′-ATGTGCATCGGCAAGTGG-3′ and 5′-CAGAAGTTGAGTTTCGGGTAG-3′ [GenBank NM_009045]. The expression of GAPDH was used to normalize cDNA levels. The PCR products were also analyzed by melting curve analysis to ascertain the specificity of amplification.

### Measurement of HMGB1, RAGE, cytokines and hydroperoxides

The levels of HMGB1 and RAGE were measured using commercially available enzyme-linked immunosorbent assay kits (HMGB1: Shino-test, Kanagawa, Japan; RAGE: R&D Systems, Minneapolis, MN, USA). Interleukin 6 (IL-6), TNF-α and chemokine (C-X-C motif) ligand 1 (CXCL-1) were measured with a Mouse Cytokine/Chemokine-Magnetic Bead Panel (Millipore, Billerica, MA, USA) in a Luminex 100 system (Millipore). The serum concentration of hydroperoxides (whole oxidant capacity of serum against N,N-diethylparaphenylene-diamine in acidic buffer) was measured as described previously [27]. The measurement unit was CARR U. It has been previously established that 1 CARR U corresponds to 0.08 mg hydrogen peroxide/dL [30].

### Statistical analysis

Data are expressed as the mean ± SEM. Comparisons were performed with the Mann–Whitney *U* test using Prism 6.0 software (GraphPad Software, San Diego, CA, USA). The *p* value of the difference in survival was determined by the log-rank (Mantel-Cox) test. *P* <0.05 was considered statistically significant.

## Results

### Anti-HMGB1 mAb significantly improves survival of H1N1-infected mice but does not affect propagation of influenza virus in the lung

**Fig. 1:**
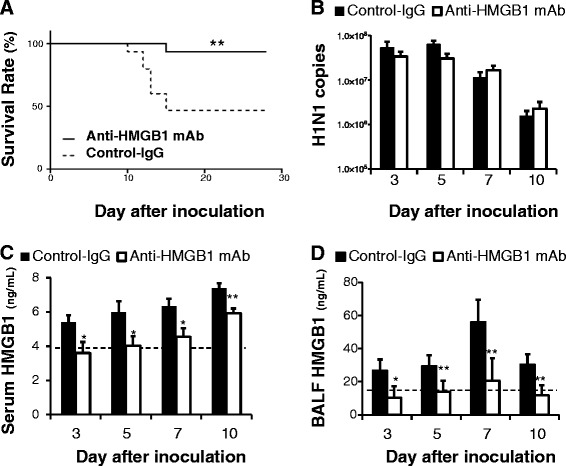
Effects of anti-high mobility group box 1 (*anti-HMGB1*) monoclonal antibody (*mAb*) treatment on survival, viral load and HMGB1 level after H1N1 inoculation. **a** Survival rate: 8 mice (53.3 %) in the control group (n = 15 mice, *broken line*) died between days 10 and 15. In the anti-HMGB1 mAb group (n = 15 mice, *solid line*), one (6.7 %) died on day 15; **p <0.01 vs control group by log-rank (Mantel-Cox) test. **b.** Viral load in the lung. Data represent the mean (± SEM) of 5 to 10 mice. There were no significant differences in viral load at any time point examined. HMGB1 levels in serum (**c**) and bronchoalveolar lavage fluid (*BALF*) (**d**). *Dotted line* indicates the normal basal level. Data represent the mean (± SEM) of 5 to 10 mice; *p <0.05 and **p <0.01 vs control by Mann–Whitney *U* test

Of the mice inoculated with 100 pfu (1 MLD_50_) influenza H1N1 virus and treated with anti-HMGB1 mAb (2 mg/kg, intravenously), 93.3 % were protected from influenza-induced death, whereas 53.3 % of infected mice administered control mAb died (Fig. 1a).

Subsequently, the viral load was determined in lung homogenates. Because the mice began to die on day 10, time points of days 3, 5, 7 and 10 after inoculation were selected for the following measurements. There was no difference in the number of viral M RNA copies in the lung at any of the time points examined between anti-HMGB1 mAb-treated mice and control mAb-treated mice upon H1N1 infection (Fig. 1b). After H1N1 infection, anti-HMGB1 mAb-treated mice had significantly lower levels of HMGB1 both in serum and BALF at all of the time points examined compared with the control mice (Fig. 1c, d).

### Anti-HMGB1 mAb significantly reduces pulmonary injury with suppression of neutrophil infiltration in the lung after H1N1 inoculation

**Fig. 2:**
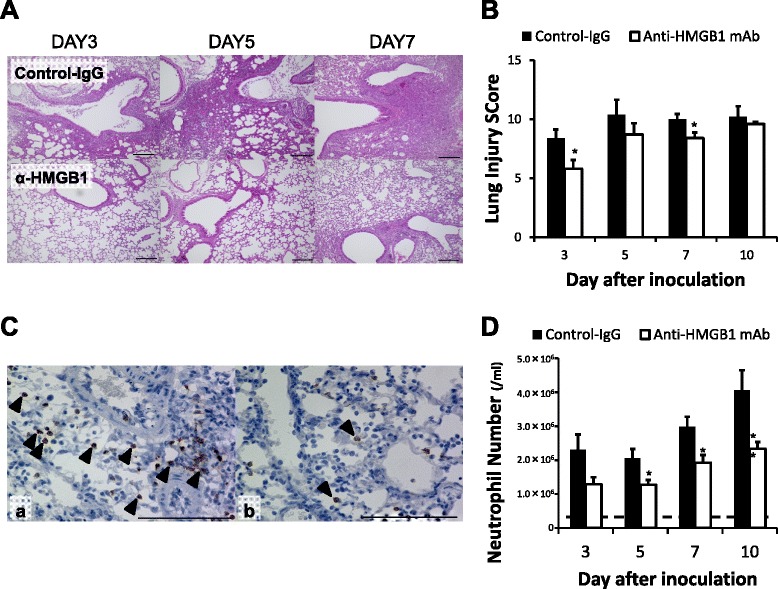
Effects of anti-high mobility group box 1 (*anti-HMGB1*) monoclonal antibody (*mAb*) treatment on lung histology after H1N1 inoculation. **a.** Photomicrographs of lung tissue samples stained with hematoxylin and eosin on days 3, 5 and 7 after H1N1 inoculation. These are representative of five independent experiments. Control mAb group lung tissue showed aggravating diffuse alveolar and interstitial edema, inflammatory cellular infiltration, hemorrhage and bronchiolitis (*upper row*). In the anti-HMGB1 mAb group lung tissue, these features were less severe (*lower row*). Scale bar = 100 μm. **b.** Lung injury scores. Data represent the mean (± SEM) of five independent experiments; *p <0.05 and **p <0.01 vs control by Mann–Whitney *U* test. **c.** Photomicrographs of lung tissue samples stained with granulocyte-differentiating antigen (Gr-1) on day 10 after H1N1 inoculation. These are representative of five independent experiments: (a) H1N1 inoculation, control group; and (b) H1N1 inoculation, anti-HMGB1 mAb group. *Arrowheads* indicate Gr-1 expressing, positively stained (*brown*) cells. Scale bar = 100 μm. **d.** Neutrophil numbers in the bronchoalveolar lavage fluid. *Dotted line* indicates the normal basal level. Data represent the mean (± SEM) of 10 mice; *p <0.05 and **p <0.01 vs control by Mann–Whitney *U* test

H1N1-inoculated control mice presented with diffuse edema, inflammatory cellular infiltration of the alveoli and interstitium of the lung, hemorrhage, and thickened airways. Treatment with anti-HMGB1 mAb attenuated the histopathological changes evident in the lung (Fig. 2a). Additionally, anti-HMGB1 mAb significantly reduced the lung injury score compared with control mice at days 3 and 7 after H1N1 inoculation (Fig. 2b). Histologically, influenza virus inoculation increased neutrophil infiltration in the lung, although anti-HMGB1 mAb treatment attenuated this effect (Fig. 2c). The neutrophil number in the BALF increased in both the anti-HMGB1 mAb and control groups; however, the neutrophil number remained significantly lower in mice treated with anti-HMGB1 mAb compared with the control mAb at days 5, 7 and 10 after H1N1 inoculation (Fig. 2d). These results indicated that anti-HMGB1 mAb treatment significantly suppressed neutrophil infiltration in the lungs of H1N1-inoculated mice.

### Anti-HMGB1 mAb inhibits the release of IL-6, TNF-α and CXCL-1, and attenuates RAGE expression in the lung after H1N1 inoculation

**Fig. 3:**
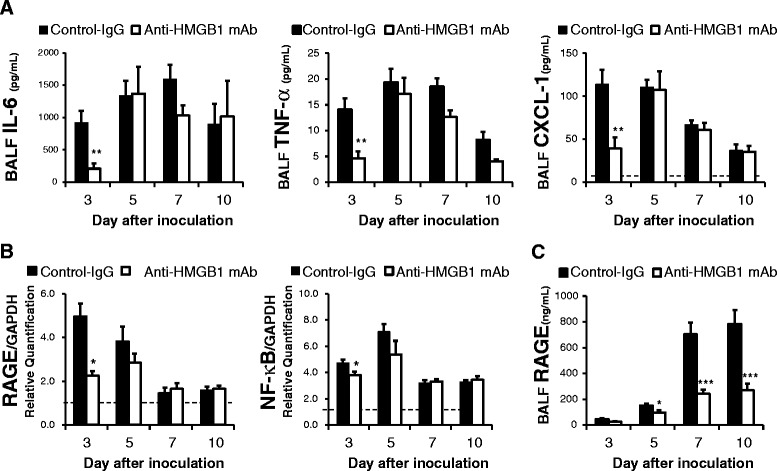
Effects of anti-high mobility group box 1 (*anti-HMGB1*) monoclonal antibody (*mAb*) on cytokines in the bronchoalveolar lavage fluid (*BALF*) and the expression of receptor for advanced glycation end products (*RAGE*) and NF-κB in the lungs after H1N1 inoculation. **a.** Cytokine concentration in the BALF. Data represent the mean (± SEM) of 10 mice. *Dotted line* indicates the normal basal level. IL-6 and TNF-α were not detected in the BALF from normal mice; *p <0.05 and **p <0.01 vs control by Mann–Whitney *U* test. **b.** RAGE and NF-κB (p65) mRNA expression in the lungs of H1N1-inoculated mice. The results were normalized to the expression of glyceralaldehyde-3-phosphate dehydrogenase (GAPDH) mRNA. The basal expression level of normal mice was calibrated as 1.0 (*dotted line*). Data represent the mean (± SEM) of 5 to 10 mice; *p <0.05 vs control by Mann–Whitney *U* test. **c.** RAGE concentration in BALF. The normal basal level was 8.3 ng/mL. Data represent the mean ± SEM of 10 mice; *p <0.05 and ***p <0.001 vs control by Mann–Whitney *U* test

Investigation of the BALF demonstrated that anti-HMGB1 mAb-treated mice had significantly lower production of IL-6, TNF-α and CXCL-1 on day 3 after H1N1 inoculation compared with control mice (Fig. 3a).

Reverse-transcription PCR in the lung homogenates showed that anti-HMGB1 mAb-treated mice had significantly attenuated *RAGE* and *NF-κB (p65)* expression on day 3 after virus inoculation compared with control mice (Fig. 3b). In addition to mRNA analysis, we measured RAGE protein levels in BALF from anti-HMGB1 mAb-treated mice and control mice after inoculation of H1N1 by an enzyme-linked immunosorbent assay. Anti-HMGB1 mAb-treated mice had significantly lower production of RAGE at days 5, 7 and 10 after H1N1 inoculation (Fig. 3c).

### Anti-HMGB1 mAb attenuates oxidative stress after H1N1 inoculation

**Fig. 4:**
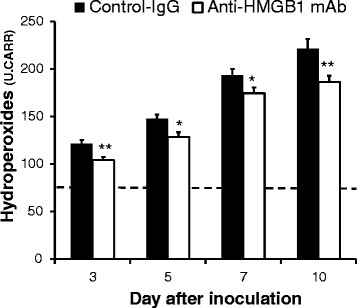
Effect of anti-high mobility group box 1 (*anti-HMGB1*) monoclonal antibody (*mAb*) treatment on hydroperoxides in serum after H1N1 inoculation. Data represent the mean (± SEM) of 10 to 15 mice. *Dotted line* indicates the normal basal level; *p <0.05 and **p <0.01 vs control by Mann–Whitney *U* test

The serum concentration of hydroperoxides was also significantly lower in anti-HMGB1 mAb-treated mice compared with control mice at all of the time points examined (Fig. 4). This result indicated that anti-HMGB1 mAb treatment attenuates the oxidative stress that is observed in H1N1-inoculated mice.

## Discussion

In the battle against severe influenza, clinicians are beginning to recognize that it is the immune response of their patient that should be modulated. That is, the involvement of inflammatory mediators in the pathogenesis of influenza has been recognized as a major issue [31]. After HMGB1 was first shown to have an additional function as a late mediator of endotoxin lethality [14], it has since been revealed as a protein with inflammatory cytokine activity in the pathogenesis of influenza [32–34], as well as in many other inflammatory diseases. Recently, HMGB1 has attracted the attention of many researchers as a therapeutic target for the treatment of various diseases [35]. Several researchers have already investigated HMGB1 and influenza and suggested the anti-influenza effects of different inhibitory agents of HMGB1, including ethyl pyruvate [36] and some Chinese herbs [37].

In this study, we evaluated the therapeutic effects of anti-HMGB1 mAb on severe H1N1-induced pneumonia in mice. We initially established an influenza-induced mouse model of pneumonia with 50 % lethality. Subsequently, 2 mg/kg of anti-HMGB1 mAb was administered by intravenous injection in triplicate after virus inoculation. The results demonstrated that systemic suppression of HMGB1 to its normal basal level by anti-HMGB1 mAb could protect against severe H1N1-induced pneumonia with almost complete survival. We also showed that anti-HMGB1 mAb attenuated RAGE expression, suggesting that RAGE plays an important role in the pathophysiological mechanism of anti-HMGB1 mAb.

Hou et al. recently reported the effects of a rabbit anti-HMGB1 polyclonal antibody against H5N1 influenza infection [32]. However, no report has identified the protective mechanism of anti-HMGB1 antibody against H1N1 influenza infection. Our findings revealed the effectiveness of anti-HMGB1 mAb against influenza infection, with a lower dose administered intravenously compared with the study by Hou et al. [32]. The antibody used in our study was monoclonal and recognized the c-terminal sequence of HMGB1. Using this anti-HMGB1 mAb, we have previously demonstrated its beneficial effects on different types of inflammatory disease, such as ischemic and traumatic brain injury [25, 26]. The primary structure of HMGB1 is conserved, with 99 % amino acid sequence homology between rodent and human. Therefore, the results in our study may be applicable to influenza infection in humans. Moreover, we have also developed a humanized anti-HMGB1 mAb (data not shown). Consequently, this study is a significant step forward in the clinical application of anti-HMGB1 mAbs for a diverse range of inflammatory diseases in humans, including tissue injury.

Cytokines and chemokines contribute to the overall pathology of lung injury, and several have been well documented in H1N1-induced pneumonia [33, 38–40]. We found that in H1N1-inoculated mice, anti-HMGB1 mAb significantly suppressed the local production of IL-6 and TNF-α, key cytokines orchestrating the pathophysiology of highly virulent influenza strains [11, 41]. IL-6 is rapidly released during the acute phase of influenza infection and its elevated levels are associated with disease severity triggered by H1N1 infection [42]. TNF-α has been shown to correlate with morbidity and mortality in influenza-infected subjects [11, 43]. Moreover, the CXCL-1 level was also significantly suppressed in mice treated with anti-HMGB1 mAb. CXCL-1 is a chemokine that directs the trafficking of circulating neutrophils to sites of inflammation or injury [44]. These effects on cytokines and chemokines help to explain our observations of a decreased local inflammatory response, attenuated infiltration of neutrophils and improved survival after H1N1 inoculation in anti-HMGB1 mAb-treated mice.

Our key finding is that treatment with anti-HMGB1 mAb resulted in suppressed expression of RAGE. RAGE is the primary binding receptor for HMGB1, and the interaction of RAGE and HMGB1 induces an inflammatory response via NF-κB activation [13]. Van Zoelen et al. [23] previously reported the importance of RAGE in the pathogenesis of influenza-induced pneumonia. Pulmonary RAGE upregulation was associated with influenza-induced pneumonia, and RAGE-deficient mice showed increased resistance to influenza-induced pneumonia. Additionally, HMGB1-induced signaling can result in the expression of RAGE via a positive feedback loop [45]. Therefore, treatment with anti-HMGB1 mAb itself could restrict RAGE expression in H1N1-induced pneumonia by blocking HMGB1-induced signaling, resulting in suppression of the inflammatory response.

Interestingly, however, the attenuation of *RAGE* expression was seen only in the early phase of infection (day 3) in anti-HMGB1 mAb-treated mice, although HMGB1 level was suppressed significantly in both serum and BALF at all time points. Correspondingly, there was no significant difference in the expression of NF-κB between the two groups in the later phase of infection (days 7 and 10). These findings help to explain the observation that few cytokines differed significantly in expression between the two groups in the later phase of infection. Soluble RAGE (sRAGE) might provide a key to answer this paradox. We found in this study that the RAGE level in BALF, which should be sRAGE, was significantly higher in control mice than in anti-HMGB1 mAb-treated mice. Without a transmembrane domain, sRAGE is reported to circulate out of the cell and act as a decoy by preventing ligands, including HMGB1, from binding to RAGE, therefore functioning as a negative feedback on RAGE interactions with its ligands [46, 47]. The hyper-expression of sRAGE in BALF might result in decreased cytokine levels. Further research on the downstream signaling pathways of the HMGB1-RAGE axis in influenza-induced pneumonia is warranted to clarify these speculations.

TLR4 signaling is involved in influenza infection [48]. In addition to RAGE, TLR4 is one of the receptors for HMGB1. Therefore, it is possible that neutralization of HMGB1 may affect TLR4 expression, leading to an altered cytokine response in the lungs. TLR4-mediated HMGB1 signaling may induce deleterious effects of HMGB1. We therefore measured the levels of TLR4 expression in the lungs after anti-HMGB1 mAb treatment. As a result, neutralization of HMGB1 did not affect TLR4 expression in the lungs compared with the control (Additional file 1). Thus, it is likely that TLR4 signaling is not directly involved in the altered cytokine response and lung pathology after anti-HMGB1 mAb treatment. We believe that RAGE-mediated HMGB1 signaling is important in this model. To test this hypothesis, a specific RAGE antibody could be used or a small interfering RNA specific for RAGE could be employed. It has been shown that mice with a RAGE deficiency were protected against influenza virus infection [23]. Further studies will be necessary to address these points.

Another key finding of this study is that anti-HMGB1 mAb treatment attenuated the serum concentration of hydroperoxides in H1N1-inoculated mice. This finding indicates that treatment with anti-HMGB1 mAb might contribute to a comprehensive suppression of not only local cytokines and chemokines but also systemic oxidative stress. Our group recently reported that administration of the redox-active protein thioredoxin-1 ameliorated H1N1-induced pneumonia in mice via its antioxidative properties, suggesting that an antioxidative strategy may be a key therapeutic regimen for influenza-induced pneumonia [27]. Therefore, it is also important to assess the oxidative stress response in influenza-induced pneumonia [49]. However, our findings warrant further study as we have not yet studied the antioxidative mechanism of the anti-HMGB1 mAb.

Although severe viral pneumonia tends to be rare during outbreaks of seasonal influenza, many cases of primary viral pneumonia were observed in the recent influenza pandemic, especially in the young [50, 51]. Even after the 2009 H1N1 pandemic, the world faces the rising burden of viral respiratory infections, including highly pathogenic avian influenza, severe acute respiratory syndrome-associated coronavirus and Middle East respiratory syndrome coronavirus [52]. Acute respiratory distress syndrome caused by these new viruses is an immediate challenge. HMGB1 was also reported to be associated with the pathogenesis of acute respiratory distress syndrome [16]. Given the limited benefit of anti-viral drugs, anti-HMGB1 mAb, which provides a protective effect against the host immunological response, shines new light on the treatment of emerging viral infections.

## Conclusions

Intravenous administration of anti-HMGB1 mAb significantly improved the survival rate and attenuated lung histopathological changes in a murine model of influenza-induced pneumonia. The protective effects of anti-HMGB1 mAb might be explained by its blockade of the interaction between HMGB1 and RAGE, a key mechanism in the initiation of inflammatory and oxidative responses. These results suggest that anti-HMGB1 mAb represents a possible therapeutic pharmacological strategy for severe influenza-induced pneumonia in humans.

## Key messages

- Intravenous administration of anti-HMGB1 monoclonal antibody significantly improved the survival rate and attenuated lung histopathological changes in a murine model of influenza-induced pneumonia.
- Intravenous anti-HMGB1 monoclonal antibody inhibited systemic and local HMGB1 levels and suppressed inflammatory cytokine/chemokine expression and oxidative stress, which were all observed in H1N1-inoculated mice.
- The expression of receptor for advanced glycation end products (RAGE) was attenuated by anti-HMGB1 monoclonal antibody treatment.

## Additional file

Additional file 1:
**Effects of anti-high mobility group box 1 (anti-HMGB1) monoclonal antibody (mAb) treatment on the expression of toll-like receptor 4 (TLR4).** The results were normalized to the expression of glyceralaldehyde-3-phosphate dehydrogenase (GAPDH) mRNA. The basal expression level of normal mice was calibrated as 1.0 (*dotted line*). Data represent the mean (± SEM) of 5 to 10 mice. The sense and antisense primers used for the analysis of the expression of TLR4 were as follows: 5′-GCACTGTTCTTCTCCTGCC-3′ and 5′-GTTTCCTGTCAGTATCAAG-3′ [GenBank NM_021297]. There was no statistical difference between groups as determined by the Mann–Whitney *U* test.

## Abbreviations

(s)RAGE: (soluble) receptor for advanced glycation end products
BALF: bronchoalveolar lavage fluid
cDNA: complementary deoxyribonucleic acid
CXCL-1: chemokine (C-X-C motif) ligand 1
GAPDH: glyceralaldehyde-3-phosphate dehydrogenase
HMGB1: high mobility group box 1
IL-6: interleukin 6
mAb: monoclonal antibody
MLD_50_: 50 % mouse lethal dose
NF-κB: nuclear factor kappa B
pfu: plaque-forming unit
SEM: standard error of the mean
TLR4: toll-like receptor 4
TNF-α: tumor necrosis factor alpha
