# Supplementary figures and images for: Anti-high mobility group box-1 monoclonal antibody treatment provides protection against influenza A virus (H1N1)-induced pneumonia in mice
Source: Crit Care. 2015 Jun 11;19(1):249. doi: 10.1186/s13054-015-0983-9 (PMC4490661; doi:10.1186/s13054-015-0983-9)

## Slide 1
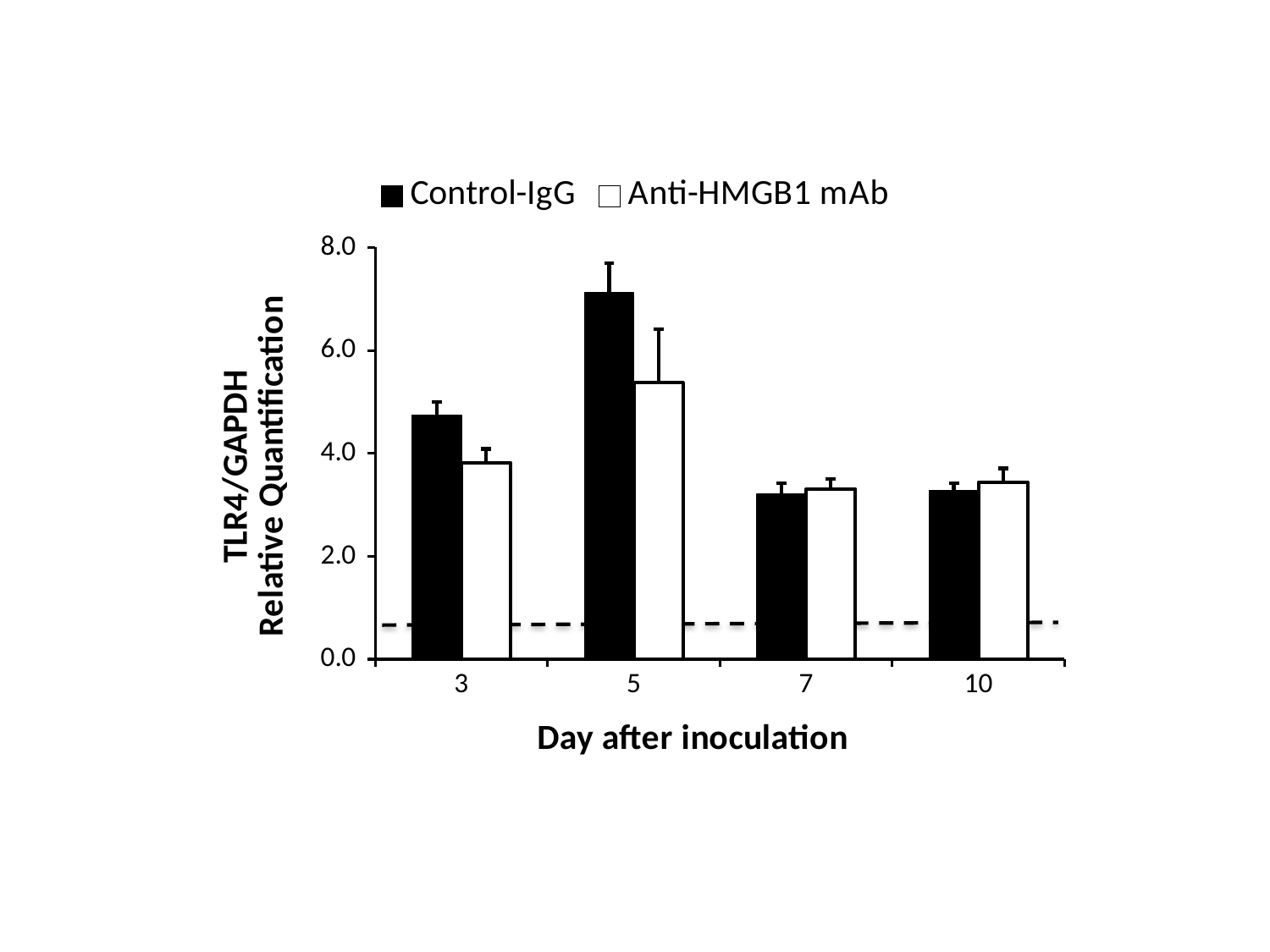

### Chart
| Category | Control-IgG | Anti-HMGB1 mAb |
|---|---|---|
| 3.0 | 4.740533333333333 | 3.816545454545454 |
| 5.0 | 7.118615384615386 | 5.372375 |
| 7.0 | 3.209466666666666 | 3.307266666666667 |
| 10.0 | 3.27025 | 3.439727272727273 |

Supplement: Additional file 1: — Effects of anti-high mobility group box 1 (anti-HMGB1) monoclonal antibody (mAb) treatment on the expression of toll-like receptor 4 (TLR4). The results were normalized to the expression of glyceralaldehyde-3-phosphate dehydrogenase (GAPDH) mRNA. The basal expression level of normal mice was calibrated as 1.0 (dotted line). Data represent the mean (± SEM) of 5 to 10 mice. The sense and antisense primers used for the analysis of the expression of TLR4 were as follows: 5′-GCACTGTTCTTCTCCTGCC-3′ and 5′-GTTTCCTGTCAGTATCAAG-3′ [GenBank NM_021297]. There was no statistical difference between groups as determined by the Mann–Whitney U test. [file 13054_2015_983_MOESM1_ESM.pptx]
